# A year of terror and a century of reflection: perspectives on the great influenza pandemic of 1918–1919

**DOI:** 10.1186/s12879-019-3750-8

**Published:** 2019-02-06

**Authors:** Michaela E. Nickol, Jason Kindrachuk

**Affiliations:** Laboratory of Emerging and Re-Emerging Viruses, Department of Medical Microbiology, University of Manitoba, 523-745 Bannatyne Avenue, Winnipeg, MB R3E 0J9 Canada

**Keywords:** Influenza, Pandemic, Pneumonia, Therapeutics, Preparedness, 1918 Spanish flu

## Abstract

**Background:**

In the spring of 1918, the “War to End All Wars”, which would ultimately claim more than 37 million lives, had entered into its final year and would change the global political and economic landscape forever. At the same time, a new global threat was emerging and would become one of the most devastating global health crises in recorded history.

**Main text:**

The 1918 H1N1 pandemic virus spread across Europe, North America, and Asia over a 12-month period resulting in an estimated 500 million infections and 50–100 million deaths worldwide, of which ~ 50% of these occurred within the fall of 1918 (Emerg Infect Dis 12:15-22, 2006, Bull Hist Med 76:105-115, 2002). However, the molecular factors that contributed to the emergence of, and subsequent public health catastrophe associated with, the 1918 pandemic virus remained largely unknown until 2005, when the characterization of the reconstructed pandemic virus was announced heralding a new era of advanced molecular investigations (Science 310:77-80, 2005). In the century following the emergence of the 1918 pandemic virus we have landed on the Moon, developed the electronic computer (and a global internet), and have eradicated smallpox. In contrast, we have a largely remedial knowledge and understanding of one of the greatest scourges in recorded history.

**Conclusion:**

Here, we reflect on the 1918 influenza pandemic, including its emergence and subsequent rapid global spread. In addition, we discuss the pathophysiology associated with the 1918 virus and its predilection for the young and healthy, the rise of influenza therapeutic research following the pandemic, and, finally, our level of preparedness for future pandemics.

## Background

Influenza viruses have posed a continual threat to global public health since at least as early as the Middle Ages, resulting in an estimated 3–5 million cases of severe illness and 291,243–645,832 deaths annually worldwide, according to a recent estimate [1]. Regional influenza epidemics occur on an annual basis, resulting in millions of illnesses and hospitalizations despite intensive vaccination and awareness programs [2, 3]. Moreover, influenza pandemics arise sporadically due to the introduction of an antigenically-distinct influenza A virus within a population, which can result in devastating effects on global public health and healthcare networks. The emergence of influenza subtype H1N1 in 1918, which ultimately resulted in an estimated 50–100 million deaths worldwide, would forever change the course of human history and will be discussed in detail in the following sections [4–6]. The aims of this short review are to discuss: i) the emergence and spread of the 1918 virus; ii) the unique severity of disease in young, healthy individuals; and iii) the subsequent influence of the pandemic on influenza virus therapeutic and future preparedness.

## Main text

### General influenza epidemiology

It is postulated that 10% of the worldwide population is infected by an influenza virus each year, resulting in a total economic burden of $87.1 billion USD [7, 8]. As a testament to the significant toll posed by influenza on public health and healthcare systems, the US Centers for Disease Control and Prevention (CDC) estimated that from 2010 to 2015, influenza infections resulted in 9.23–35.6 million illnesses and 139,000–707,000 hospitalizations annually in the US alone [9]. It has been suggested that children are likely the primary transmitters of influenza [10]. Lethal influenza infections are primarily associated with high risk populations, including infants (< 1 year), the elderly (> 65 years), and individuals with pre-existing comorbidities, including chronic respiratory abnormalities, cardiac disease, immunodeficiency, and pregnancy [11, 12]. Mortality in children and young adults is generally low [3]. Symptoms manifest as a sudden high fever, headache, pharyngitis, cough, myalgia, nausea, vomiting, and fatigue, which generally resolve within 7 days in healthy adults [11, 13]. Severe and/or lethal disease is typically associated with viral pneumonia or secondary bacterial infections in the lower respiratory tract [3].

### A history of influenza pandemics

To be considered a pandemic, an influenza virus must: i) spread globally from a distinct location with high rates of infectivity resulting in increased mortality; and ii) the hemagglutinin (HA) cannot be related to influenza strains circulating prior to the outbreak nor have resulted from mutation [14, 15]. It should also be appreciated that prior to the first isolation of a human influenza virus in 1933, the cause of influenza outbreaks and pandemics could only be inferred based on physiological symptoms of disease, along with the speed and breadth at which illness was spread [15].

As early as 412 BC, Hippocrates, the father of modern medicine, described the first known account of an influenza-like illness in his sixth “Book of Epidemics” [16, 17]. Here, he recounted an annual recurring upper respiratory tract infection characterized by pharyngitis, coryza, and myalgia which peaked around the winter solstice [18]. This seasonal epidemic occurred in Perinthus, a northern port town located in what is now Turkey, and is referred to as the “Cough of Perinthus” [16]. It has been suggested that potential pandemics may have occurred in 1510 and 1557; however, it is unanimously agreed that the first documented influenza pandemic occurred in 1580, resulting in high morbidity [15, 19]. The virus originated in Asia, before spreading to Africa, and then simultaneously spreading from both continents to Europe. It reportedly spread across the entire European continent within 6 months, before eventually reaching the Americas [19, 20]. Two pandemics were recorded in the eighteenth century. The first began in Russia in 1729, eventually moving across the entirety of Europe within 6 months and, ultimately, across the known world over the following 3 years [20–23]. The second pandemic began in China in 1781, before spreading to Russia and, subsequently, across all of Europe. Interestingly, this second pandemic had a high proclivity for young adults [24]. Two major pandemics also occurred throughout the nineteenth century. The first began in 1830 in China, with subsequent spread to Southeast Asia, Russia, Europe, and North America and had a low overall mortality rate [15, 19, 20, 23]. A second pandemic emerged in Russia in 1889 and spread rapidly to Europe and North America, circumnavigating the globe in just 4 months [25, 26]. The virus, suggested to be of subtype H3N8, reappeared at least 3 more times in successive years resulting in an estimated 1 million global fatalities [20, 23, 26, 27].

**Fig. 1:**
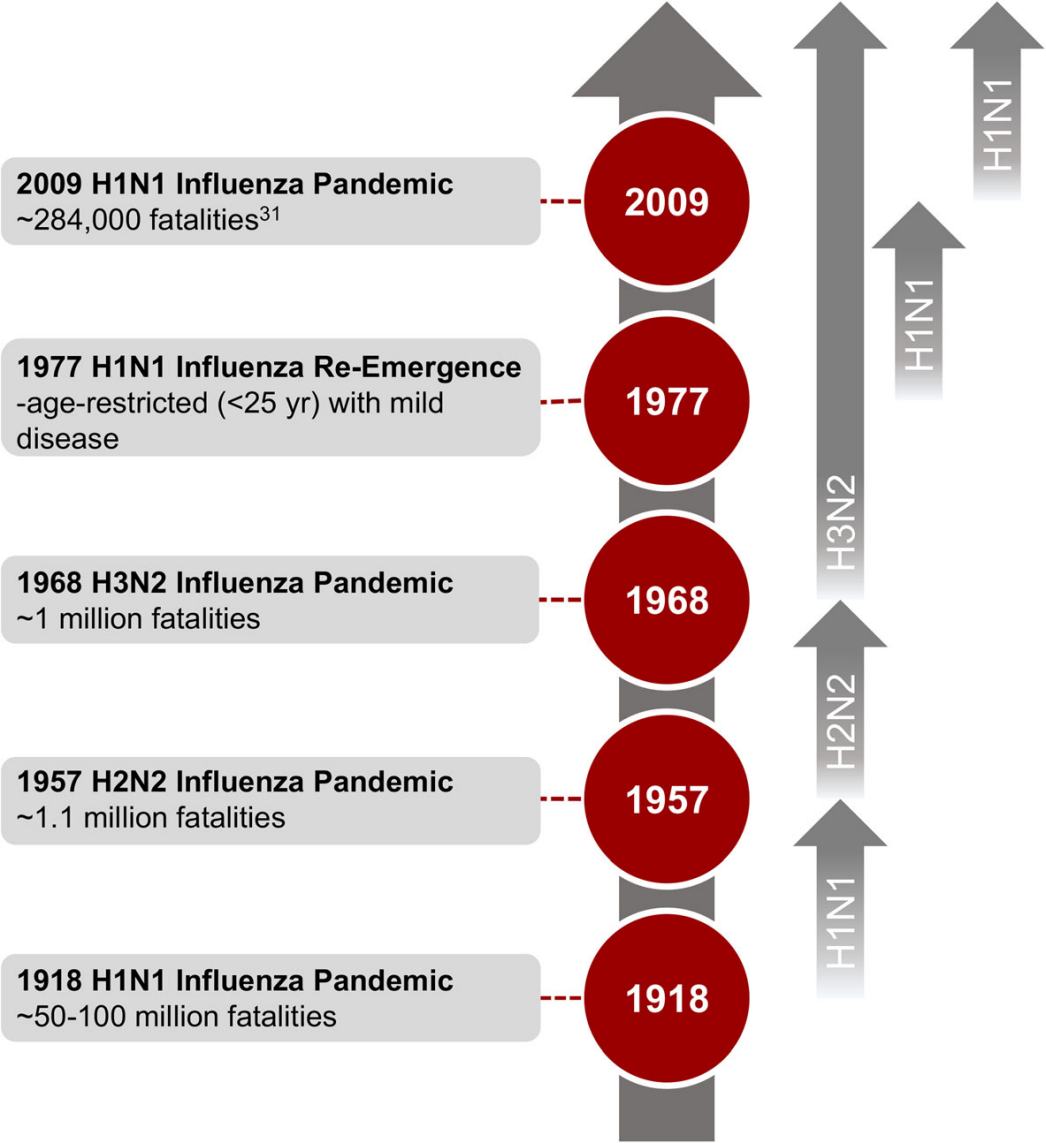
Timeline of Influenza Pandemics from 1918 Onwards. Four pandemics have occurred over the last century (1918, 1957, 1968 and 2009). Circulation of H1N1 was reinitiated in 1977 and has therefore been added to this timeline. Grey arrows designate the circulating or co-circulating strains during the interpandemic periods

Four influenza pandemics have occurred over the past century (Fig. 1). The 1918–1919 Spanish flu pandemic, subtype H1N1, resulted in an estimated 50–100 million deaths worldwide and will be discussed in detail in the following sections. The 1957–1958 Asian flu pandemic, subtype H2N2, originated in China in February 1957 and spread throughout Asia and then globally by the summer. Case fatality rates were approximately 0.67% with 1–2 million deaths worldwide [20, 28–31]. Just a decade later, the 1968–1970 Hong Kong flu pandemic, subtype H3N2, emerged in China in July 1968 and spread throughout Europe, North America, and Australia by early 1969 [25]. Although mortality rates were low, the pandemic would ultimately claim between 500,000 and 2 million lives [25]. In April 2009, the 2009–2010 swine flu pandemic, subtype H1N1, began with nearly simultaneous outbreaks in Mexico and the US, before spreading globally over the next 6 weeks. While the 2009–2010 pandemic had a low associated case fatality rate, resulting in 284,000 deaths worldwide, it had devastating effects on global economies and healthcare networks [25, 32]. Conventionally, influenza pandemics result in the extinction of previously circulating virus strains; however, this view was complicated by events in 1977. Although H1N1 was replaced by H2N2 as the circulating strain following the 1957–1958 Asian flu pandemic, a descendant of the 1918 virus “re-emerged” suspiciously in 1977, likely as a result of a man-made event, and established itself as a co-circulating strain, along with the reassortant H3N2 virus (following the 1968–1970 Hong Kong flu pandemic) [4, 33]. The suspicious “re-emergence” of a descendant of the 1918 virus in 1977 has been postulated to have been the result of a man-made event. This hypothesis has gained traction, as both the HA and NA of the re-emerged virus show incredible similarity to a 1950 reference virus, and it is unlikely that this strain was maintained in an animal reservoir for almost two decades without having undergone detectable mutation [33]. In 2009, a triple reassortment (made up of avian, swine, and human influenza genes) pandemic H1N1 jumped from pigs to humans, resulting in the co-circulation of three influenza strains [34].

### The first wave of the 1918 pandemic

One hundred years following its emergence, the origin of the 1918 pandemic influenza virus remains shrouded in mystery. The 1918 pandemic began early in the final year of the First World War. Whereas prior pandemics had spread largely along trade routes, the global context of the war enabled greater viral spread facilitated by the mass mobilisation of military personnel and civilians [25, 35]. This was further augmented by the poor health and sanitation conditions found within trenches along the frontlines of the War, facilitating disease transmission [36]. Public knowledge regarding the severity of the pandemic was hindered, as many news agencies were barred from writing about the global health threat, instead reporting solely on morale boosting subjects [37]. However, as Spain was a neutral party in the War, newspapers were able to report on the devastating effects that the 1918 pandemic virus was exhibiting in Spain. Thus, it was generally perceived that this devastating illness originated in Spain, resulting in the pandemic being incorrectly labeled as “the Spanish flu” [37].

A century following its emergence, there remains a relative paucity of knowledge regarding the ancestry and regional origin of the 1918 virus. Sequence analysis suggests that the virus was derived from an avian-like influenza virus and that all eight gene segments likely evolved in parallel [34, 38]. Analyses of influenza virus genome sequences also suggest that the initial entry of the 1918 precursor virus into human circulation began in 1915 and did not appear to have jumped directly from an avian source [4, 38, 39]. However, improved understanding regarding the emergence of the 1918 virus, as well as factors (biological, social, environmental) that contributed to viral transmission and pathogenesis, have been vital to the development of current epidemic and pandemic influenza outbreak response efforts. Descendants of the 1918 pandemic influenza virus strain have been the cause of almost every seasonal influenza A infection worldwide over the past century [4]. Additionally, each of the pandemics occurring in 1957, 1968, and 2009 were caused by descendants of the 1918 pandemic influenza virus strain, earning the 1918 viral strain the nickname “The Mother of all Pandemics” [4].

**Fig. 2:**
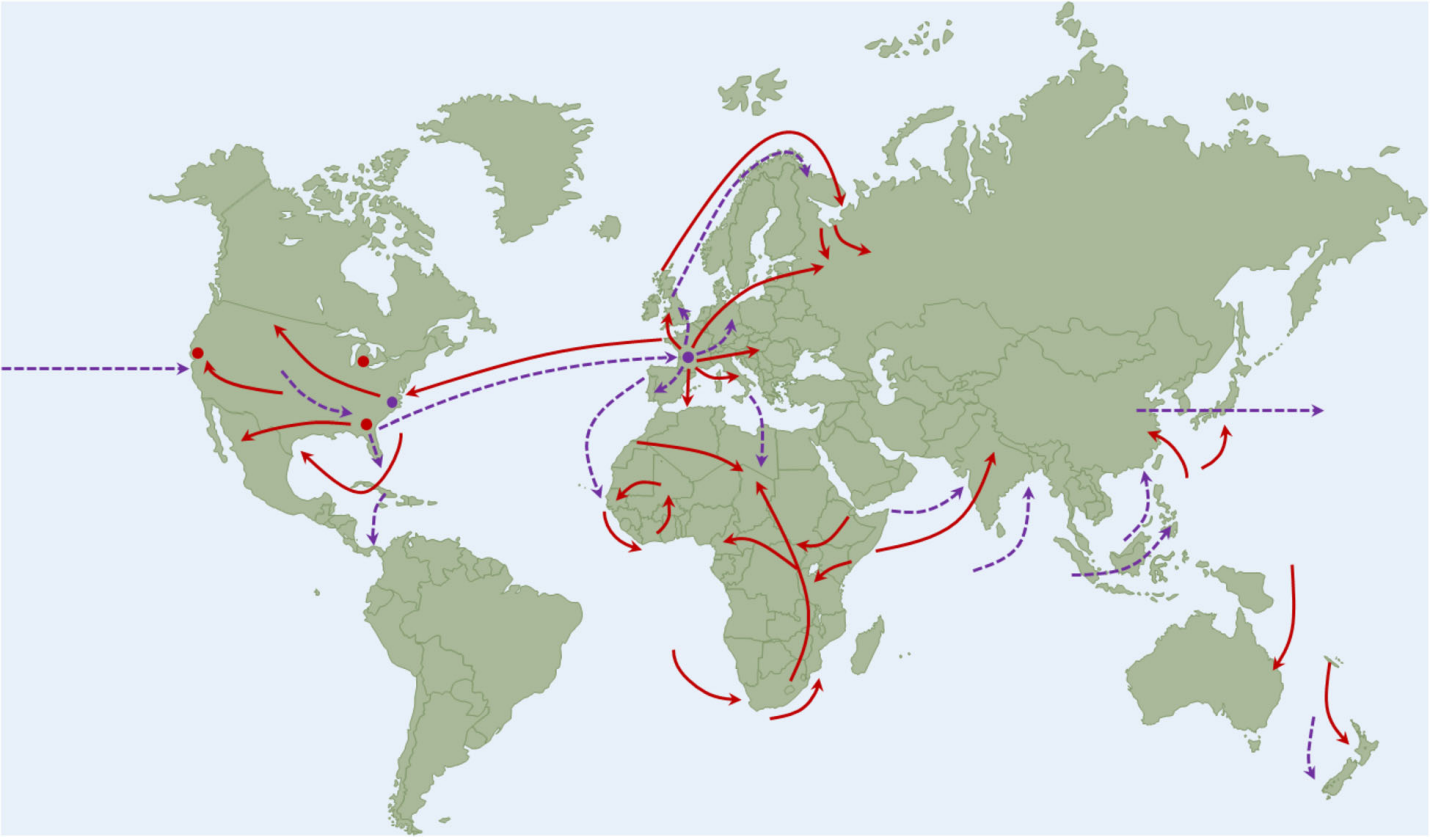
The First and Second Waves of the 1918–1919 Pandemic. First outbreaks and foci of second waves of the pandemic are labeled as red and purple circles, respectively. The lines of spread of the first and second waves of the pandemic are labeled as purple dashed lines and red solid lines, respectively. Map images were derived and/or modified from Servier Medical Arts under a Creative Commons Attribution 3.0 Unported License. Adapted from Nicholson et al. [80]

Investigations concerning the origins of the first wave of the pandemic, beginning in March 1918, have primarily focused on the US and China, though recently it has been suggested that the origin may have been an outbreak of a respiratory disease misidentified as pneumonic plague in China [15, 36, 40]. Humphries suggests that the dissemination of labourers from China to assist Allied war efforts during this outbreak resulted in the inadvertent spread of the virus to Europe [36]. From 1916 to 1918, the route of travel to Europe for the labourers included checkpoints in Singapore, Durban, Cape Town, North Africa, and Canada. Additional reports of the first wave of the virus in the spring of 1918 suggest that the pandemic originated with Chinese workers at Camp Funston, Kansas, where the workers began suffering from 2 to 3 day fevers, gastrointestinal symptoms, and general weakness [37, 41]. Within 3 weeks 1100 soldiers had been hospitalized, and thousands more had received out-patient treatment [41]. The illness was able to spread to other military camps within the US, before traversing the Atlantic Ocean via soldiers supporting Allied operations in Europe. The US Army reported that from March–May 1918, 11.8% of US soldiers were hospitalized due to this unidentified respiratory illness [41]. While illness rates were high during this initial wave, mortality rates were largely similar to seasonal outbreaks of influenza. Spain reported that the mortality rates for pneumonia and influenza was only 0.065% [37]. Although there was some acceptance that this new illness was indeed influenza, this was not generally accepted [37]. Radusin reported that although the physiological symptoms were similar to influenza, the illness was too mild and short-lasting with minimal complications for it to be influenza [37]. Infections began to subside in many regions by the early summer [41]. The generally accepted lines of spread of the first and second waves of the 1918 virus are provided in Fig. 2.

### The second and third waves of the pandemic

In mid-August of 1918, reports suggesting a second wave of this severe illness began to surface [35]. In some regions, primarily Northern Europe, the period between the end of the first wave and the beginning of the second wave was incredibly short, making the two waves almost indistinguishable [4, 42]. This second wave, occurring from September–November 1918, was responsible for the majority of illnesses and fatalities associated with the pandemic. Although the origins of the first wave continue to be debated, the origin of the second wave is generally agreed to be the harbour town of Plymouth in Southern England, which allowed the pandemic influenza virus strain to easily spread to the rest of the world [25]. Ships from Plymouth were dispatched to Freetown, Sierra Leone in August 1918, which allowed the virus to spread across the African continent [25]. New Zealand soldiers, who stopped in Freetown on their way to and from the war front in Europe, facilitated transfer of the pandemic virus to New Zealand [25]. From Plymouth, the virus also spread to Boston, from which it was able to disseminate across the rest of North America resulting in > 1 million fatalities over the ensuing four months [5, 25]. This second wave spread globally throughout the fall of 1918 with illness seen first amongst military personnel and, subsequently, within the general population [25, 35].

The second wave of the 1918 pandemic differed from the first in that much higher morbidity and mortality rates were reported, with the majority of all fatalities associated with the pandemic occurring during this wave [4]. Ultimately, the pandemic would result in an estimated 500 million infections worldwide (~ 1/3 of the world’s population at the time) and a case fatality rate > 2.5%, more than 25 times higher than any other pandemic [4, 37]. As a testament to the severity of this second wave, during the fall of 1918, the first 4–5 pages of Spanish newspapers were filled with obituaries of those who had succumbed to the pandemic virus [35]. Further, reports from Philadelphia, Pennsylvania stated that across 31 hospitals in the city, every hospital bed was occupied by patients with influenza [35]. The pandemic was especially problematic in highly isolated communities where many individuals had limited contact with prior influenza strains, thus lacking any pre-existing immunity. For example, some Inuit settlements reported case mortality rates as high as 70%, while certain communities in Africa were completely decimated [35]. Interestingly, individuals who had been infected throughout the first wave seemed to be protected against this secondary wave, and recent analyses have suggested that these individuals had up to 94% protection throughout the fall wave [4, 41].

A third and final wave of the pandemic appeared in most of the world in the early months of 1919 [4, 5, 35]. This final wave generally overlapped the first wave in terms of regional distribution; however, it seemed to spare areas where the second wave had been especially severe. Overall, morbidity rates were lower throughout this final influenza wave; however, mortality rates are believed to have been just as severe as the second wave [4, 35]. Three successive annual winter post-pandemic recurrences occurred following the third wave of the pandemic with continually decreasing mortality rates, in particular within those 20–40 years of age [43].

### Pathophysiology of the 1918 pandemic influenza virus

**Fig. 3:**
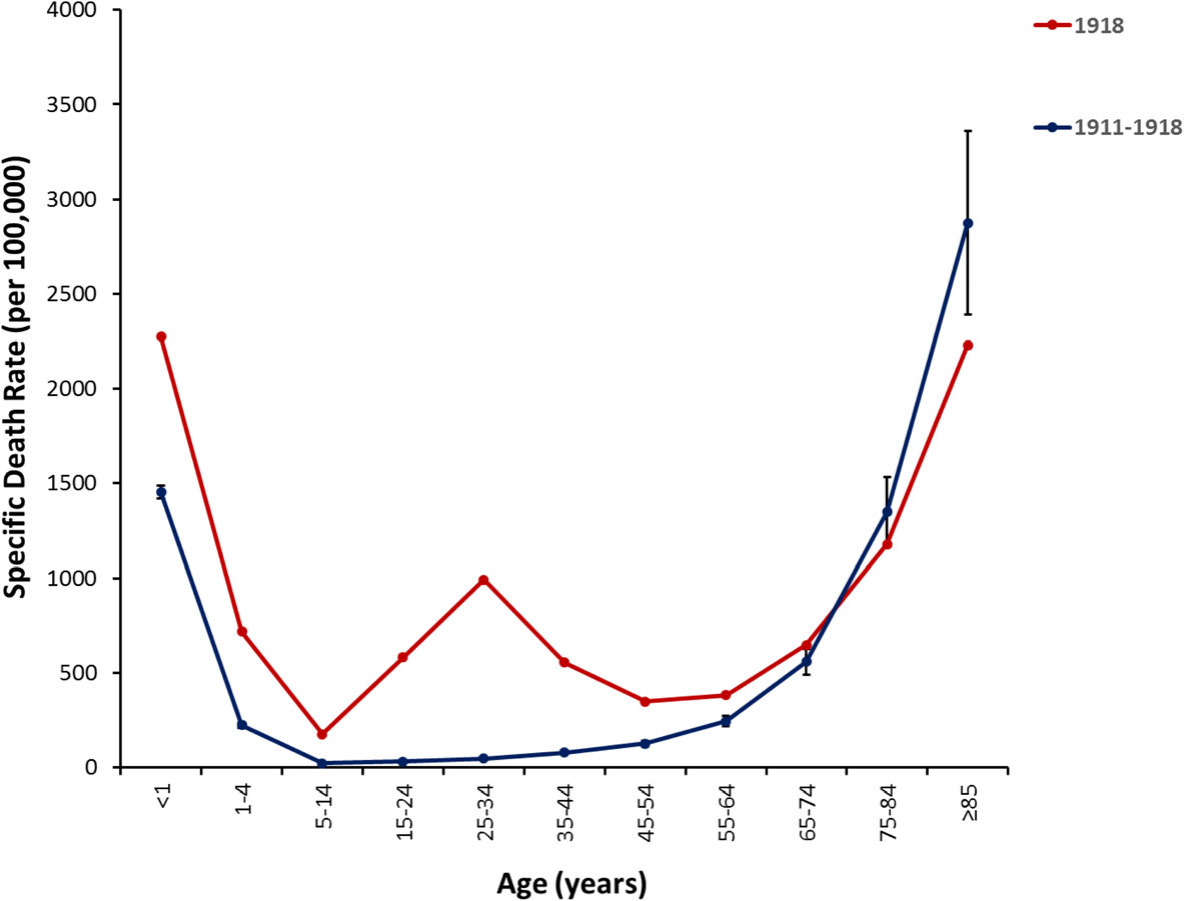
Association of Age with Influenza Mortality Prior to and During the 1918–1919 pandemic. Influenza- and pneumonia-specific mortality in the United States is plotted for 1911–1917 (blue line) and for 1918 (red line) [81, 82]. Means with standard deviations are presented for the pre-pandemic mortality curve. Adapted from Taubenberger and Morens [4]

Classically, fatal influenza infections are primarily associated with the very young (< 5 years) and the elderly (> 65 years) resulting in a characteristic “U”-shaped mortality curve (Fig. 3). Interestingly, however, the 1918–1919 H1N1 influenza pandemic mortality curve exhibits a “W”-shape due to excess mortality in young adults 20–40 years of age due to influenza-related illness. It has been postulated that the increased disease severity in young adults was likely associated with immune status due to the lack of pre-existing immunity in this population [44]. Further, more than 99% of fatal infections occurred in those < 65 years of age and nearly 50% of all influenza-related deaths during the 1918 pandemic were in those aged 20–40 years [4]. Influenza and pneumonia fatality rates in those aged 15–34 years were more than 20 times higher than in previous years and absolute risk of influenza-related death was higher in those < 65 years of age than those > 65 years old [4]. It is still not fully understood why this occurred, but it is possible that an antigenically similar influenza strain circulated prior to 1889, providing a level of protection against the novel H1N1 pandemic strain to those born prior to 1889 [4]. Additionally, archaeserological and epidemiological evidence have shown that an H3 subtype influenza virus may have been responsible for the 1889 influenza pandemic, which circulated until the emergence of the 1918 pandemic virus, leaving those individuals who had not been exposed to an H1 subtype virus highly susceptible to the pandemic virus [34]. It has also been suggested that the generation of an excessive inflammatory response (“cytokine storm”) in healthy, young adults infected with the 1918 virus may have contributed to the excess mortality seen within this age group [34]. Recent in vivo studies with the 1918 virus have shown a marked upregulation of inflammatory cytokines, along with the suppression of important antiviral immune responses [34, 45]. In addition, other influenza strains, such as fatal H5N1 infections in humans, have also been associated with the deleterious consequences of an excessive inflammatory response [46]. Ultimately, the case fatality rate was so severe in young adults during the 1918–1919 pandemic that the average life expectancy rate in the US dropped by ~ 12 years [47].

Physiological symptoms of the 1918 pandemic virus generally lasted for 7 days and were described as feeling cold, shivering, high fever, weakness, nausea, loss of appetite, pharyngitis, cough, and bloodshot eyes [35]. In some patients, a short “rebound” to normal health would occur that was followed by an aggressive recrudescence of disease and, ultimately, death [35]. Similar to the 1889 pandemic, the majority of fatal infections resulted from respiratory complications. However, it has also been demonstrated that excess influenza fatalities during the 1918–1919 pandemic were associated with an acute aggressive bronchopneumonia (including epithelial and vascular necrosis, hemorrhage, edema, and bacterial-associated variant pathology within the lungs) and a severe acute respiratory distress-like syndrome associated with severe facial cyanosis [43].

Autopsies performed on preserved lung tissues in the modern era have revealed acute pulmonary hemorrhage and secondary bacterial infections associated with pulmonary lesions in nearly all the fatal cases examined [41, 43, 47]. *Streptococcus pneumoniae* was present in many cases; however, *Staphylococcus aureus*, *Haemophilus influenzae*, and *Streptococcus pyogenes* also appeared to complicate fatal cases [48, 49]. Neutrophilic pulmonary infiltration was seen in cases of pneumococcal pneumonia, while cases of staphylococcal pneumonia were marked by multiple microabscesses infiltrated by neutrophils [48]. However, alveolar cell damage was seen in each case along with pulmonary repair and remodelling [48]. Tissues from each of the fatal cases examined had similar pathologic presentation, independent of which pandemic wave they were associated with. Despite the difference in mortality rates, each wave showed similar cellular tropism, infecting both type I and type II pneumocytes, as well as the bronchiolar respiratory epithelium [48].

### The rise of vaccines and antivirals following the 1918–1919 pandemic

A multitude of scientific and technological advances have occurred over the past century, allowing for a greater understanding of the dynamic relationship between the host and influenza viruses during infection. These advances, along with access to autopsy samples and the reconstitution of the 1918 pandemic virus, have facilitated a greater understanding of how the pandemic virus differs from other seasonal and pandemic influenza virus strains. Moreover, technological advancements following the 1918–1919 influenza pandemic virus have facilitated the development of preventative measures, including vaccines and antivirals, to limit widespread illness due to influenza infections.

The determination of the genomic sequence of the 1918 pandemic virus, and the subsequent reconstruction of the virus, has provided us with the opportunity to decipher the viral- and host-specific properties that contributed to the severity of the 1918–1919 pandemic. It has been demonstrated that in contrast to other influenza viruses, the 1918 pandemic virus is highly virulent and pathogenic in multiple animal species without prior adaptation [45, 50]. While obvious knowledge gaps remain, in particular with respect to the origin of the virus and the molecular mechanisms (host and/or viral) underlying differential pathogenesis as compared to other influenza viruses, there have been considerable advances in our understanding of the 1918 pandemic virus.

Since the isolation of the first human influenza virus in 1933, researchers have worked to develop an effective influenza vaccine [16]. Current influenza vaccines are reformulated seasonally and provide protection against circulating influenza A and B viruses [13]. The World Health Organization conducts worldwide surveillance studies throughout the year on currently circulating influenza strains, and thus recommends which strains should be included in each influenza vaccine [13]. While the seasonal influenza vaccine is approximately 60% effective, this protection is dependent on the characteristics of the individual being vaccinated, including age and overall health, as well as the match between the strains included in the vaccine formulation and currently circulating strains [13]. Individuals who have been vaccinated are generally protected from illness and provide a measure of protection for those who are not able to be vaccinated due to their age or other health issues through herd immunity [13]. There has also been increasing interest in the development of “universal” influenza vaccines designed to provide protection against a wide range of antigenically-distinct influenza viruses, including those currently in circulation and those that may emerge in the future [51]. These will not be discussed in detail as recent reviews have provided excellent discussions of this topic [51–57].

Two major classes of antivirals have emerged for therapeutic treatment of severe influenza virus infections. Adamantane antivirals target the matrix-2 (M2) surface protein, while neuraminidase (NA) inhibitors target the NA viral surface protein. Adamantane compounds were the first licensed influenza antivirals and block the M2 ion channel protein from properly functioning, thus effectively blocking membrane fusion [58, 59]. Unfortunately, adamantane antivirals are only able to target influenza A viruses limiting their application for influenza B virus infections [58]. Further, more than 90% of influenza A viruses are resistant to this class of drugs due to the high mutation rate of the virus [58, 60]. Thus, the use of NA inhibitors is recommended [60]. NA inhibitors block the NA surface protein and prevent the release of progeny virus and infection of additional cells [60]. While resistance to NA inhibitors has been observed in some influenza virus strains, they are still highly effective in the majority of patients [60]. Studies have shown that both adamantane antivirals and NA inhibitors provide protection against the 1918 virus [50].

Although outside the auspice of this commentary, it should be mentioned that advances in mechanical ventilation modalities, including non-invasive positive pressure ventilation, from the 1950s onwards, have provided an additional support mechanism for treatment of severely ill patients [34]. The routine clinical use of antibiotics in the early twentieth century also heralded a new era for combating influenza viruses. As a testament to this, excess influenza mortality declined significantly from 1942 to 1951 onwards [61–63]. However, the widespread general administration of antibiotics has resulted in an escalating public health crisis due to multi-drug resistance. This has impacted the treatment of severe influenza infections, as methicillin-resistant *S. aureus* (MRSA) is the most frequently isolated bacteria from patients with severe influenza-bacterial co-infections in the US [64, 65] and complicated up to 55% of fatalities during the 2009 pandemic [66–69].

### Influenza preparedness and lessons for the future

Although it has now been a century since the start of the Spanish flu pandemic, lessons from this global health catastrophe continue to inform modern-day pandemic preparedness. Investigations of the pandemic, including those with the reconstructed virus, have allowed researchers, as well as the global public, to understand the mechanisms that underlie pandemic emergence and escalation to public health crisis. It also allows researchers to predict the potential public health risks which may be caused by new pandemic viruses. For example, sequencing of the 1918 pandemic virus revealed similarities in the H1 protein of the 2009 pandemic virus, allowing researchers to predict that a lack of protection, and thus a high mortality rate may be seen in healthy, young adults throughout the 2009 H1N1 pandemic [45]. Thus, when vaccines were limited during the early stages of the 2009 pandemic, young adults were prioritized over the elderly, who demonstrated some degree of protection to this influenza strain, resulting in a lower mortality rate in young, healthy adults [45]. The average age for laboratory-confirmed fatalities during the 2009 pandemic was 37 years in the US, supporting this vaccine prioritization initiative [70]. Additionally, the awareness of the complications caused by secondary bacterial co-infections from the 1918 pandemic ensured that the medical community was aware of this threat throughout the 2009 pandemic, likely resulting in a reduced mortality rate due to severe influenza infections with complications [45].

However, the 2009 pandemic, albeit milder than previous pandemics in terms of overall mortality, resulted in significant strains on global healthcare networks and economies [25]. In Canada, direct healthcare costs (including hospitalizations, outpatient visits, and therapeutics) related to the 2009 pandemic have been estimated at $2 billion CAD, with $250 million CAD related directly to hospital care [71]. A computational modeling study by Smith and colleagues suggested that direct costs related to illness would be between 0.5–4.3% of GDP in the UK for pandemics ranging from low to extreme [72]. Further, the 2002–2004 severe acute respiratory syndrome outbreak resulted in ~$1 billion total GDP loss in Toronto alone [73]. This highlights the importance of pandemic preparedness beyond a healthcare-centric approach to one that also includes downstream economic effects.

The 1918–1919 pandemic resulted in incredible improvements to public health as well as scientific advances. However, our current understanding of influenza viruses, and their ability to cause illness in humans is still in its infancy in many aspects, and further underlines our inherent need for continued influenza research. The identification of key molecular determinants involved in the pathophysiology of severe influenza infections will also assist drug discovery and development strategies, including insights on appropriate timing for administration of antivirals and/or antibiotics. The development of efficacious broader-spectrum or “universal” influenza vaccines is also of incredible importance. The emergence of novel highly pathogenic avian influenza (HPAI) viruses, including H5 and H7 subtypes, are of particular concern due to their pandemic potential. Circulating HPAI viruses are of potential concern to global public health [74]. Asian lineage avian influenza A (H5N1), which circulates in fowl, is rarely found in humans but has resulted in life-threatening cases when able to establish stable lineages [74] and H7N9 has resulted in sporadic human infections in China resulting in > 1500 infections with an estimated 39% case fatality rate since 2013 [75]. Because HPAI viruses can arise from previously known low-pathogenicity viruses with only minor mutations, it is important to be vigilant concerning these potential pandemic viruses [76, 77].

## Conclusions

In spite of the public health advancements in the 100 years following the 1918–1919 pandemic, including widespread access in the developed world to an efficacious influenza vaccine, influenza viruses remain a global public health threat. This pas year, there were > 55,000 reported influenza infections, 5155 influenza-associated hospitalizations, and 303 deaths across Canada [78]. Further, during the 2016–2017 influenza season, vaccination rates in those 18–64 years of age was only 37 and 69% in those ≥65, both below the national vaccination target of 80% [79]. These data suggest that our continued vigilance against influenza must not only include a “research”-driven focus but also include public outreach and awareness campaigns that increase the general understanding of the healthcare burden associated with influenza infections.
